# Topiramate-associated bilateral anterior uveitis and angle closure glaucoma

**DOI:** 10.4103/0301-4738.71700

**Published:** 2010

**Authors:** Nibedita Acharya, Suneetha Nithyanandam, Sripathi Kamat

**Affiliations:** Department of Ophthalmology, St John’s Medical College Hospital, Sarjapur Road, Bangalore – 560 034, Karnataka, India

Dear Editor,

We congratulate the authors Senthil *et al*. for their paper on “Bilateral simultaneous acute angle closure caused by sulphonamide derivatives: A case series.”[1] With reference to their paper, we want to share our experience in the management of topiramate-associated bilateral severe uveitis with angle closure glaucoma, a rare complication of topiramate therapy. In the literature there are no reports of uveitis associated with topiramate therapy although the drug literature mentions uveitis as one of its adverse effects.[2,3]

A 49-year-old male sought treatment for severe bilateral headache, redness, and watering of eyes of 1-day duration. The patient gave history of topiramate therapy, initiated 2 weeks prior to the onset of ocular symptoms for alcohol deaddiction.

On examination, the best corrected visual acuity was <20/1200 in both eyes. There was bilateral circum-corneal congestion, severe corneal edema, and very shallow anterior chamber in both eyes. Intraocular pressure was 37 mmHg in both eyes as measured by applanation tonometry. Topical timolol 0.5% twice daily, brimonidine 0.1% three times daily, oral acetazolamide 250mg four times daily, and intravenous 20% mannitol 100 ml twice daily were administered, and topiramate was discontinued.

**Figure 1:**
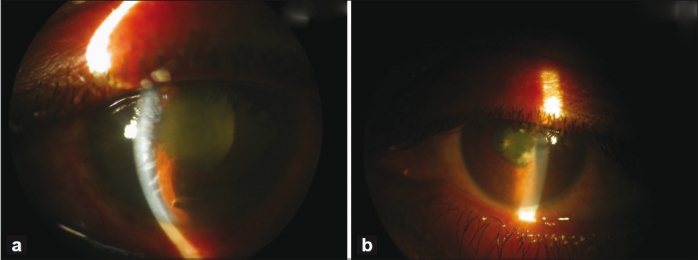
(a) Slit-lamp photograph of the right eye showing corneal edema and shallow anterior chamber, and fibrinous exudates in the anterior chamber. (b) Slit-lamp photograph of the left eye showing the shallow anterior chamber and fibrinous exudates in the anterior chamber

**Figure 2:**
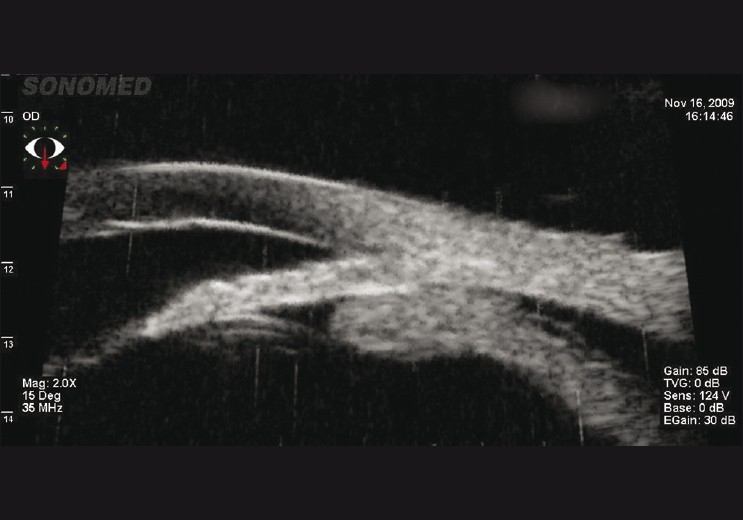
Ultrasound biomicroscopy of the right eye showing anterior rotation of the ciliary body, closed angles, and ciliochoroidal detachment

Twenty-four hours later corneal clarity improved, revealing severe nongranulomatous anterior uveitis with grade 4 cells and flare in both eyes [Fig. 1a and b]. Diffuse fine-to-medium keratic precipitates were present on the posterior surface of the cornea. Ultrasound biomicroscopy (UBM) of the right eye revealed increased central corneal thickness and severe anterior chamber reaction, the angles were closed over 360°, caused by anterior rotation of the ciliary body and forward shift of the iris-lens diaphragm [Fig. 2]. In the right eye, there was demonstrable choroidal effusion and peripheral choroidal detachment. Similar but less severe UBM findings were detected in the left eye.

Topical and systemic steroids along with topical atropine were administered for severe uveitis, while continuing antiglaucoma measures. After 1 week of treatment, the signs gradually reduced and the visual acuity improved to 20/120 in the right eye and to 20/30 in the left eye.

All medications were gradually tapered and stopped over 6 weeks. At 6 weeks the best corrected visual acuity was 20/40 in the right eye and 20/20 in the left eye. At 6-month follow-up, there was a residual membrane over the lens with 180° posterior synechiae in the right eye, with the left eye being normal. In both eyes IOP was normal.

In the literature, there are many reports of topiramate-induced myopia and angle closure glaucoma.[1,4–6] However, to the best of our knowledge there is no report of topiramate-induced uveitis in the English language literature. Our patient had bilateral severe uveitis with angle closure glaucoma, which was temporally associated with the drug usage.

Causality assessment using Naranjo’s algorithm and WHO Probability Scale was done. The pre-existing case reports, the presence of a temporal association between the administration of the drug and the onset of the adverse drug reaction (ADR), and the resolution of the ocular pathology following dechallenge puts this ADR under the “probable” category with a Naranjo’s score of 7.[7] As the initial ADR was severe, with residual visual loss and posterior synechiae formation in the right eye, rechallenge was not done. Myopia, angle closure glaucoma, and uveitis can be considered as a progressively increasing severity of the idiosyncratic reaction to topiramate and other sulphonamide group drugs.

The above case highlights the importance of increasing the awareness of this rare, idiosyncratic ADR of topiramate and the need for timely intervention to avoid irreversible visual loss.
